# Qishen Yiqi Dripping Pill Protects Against Diabetic Nephropathy by Inhibiting the Wnt/β-Catenin and Transforming Growth Factor-β/Smad Signaling Pathways in Rats

**DOI:** 10.3389/fphys.2020.613324

**Published:** 2021-02-19

**Authors:** Qian Zhang, Xinhua Xiao, Jia Zheng, Ming Li, Miao Yu, Fan Ping, Tong Wang, Xiaojing Wang

**Affiliations:** Key Laboratory of Endocrinology, Ministry of Health, Department of Endocrinology, Peking Union Medical College Hospital, Peking Union Medical College, Chinese Academy of Medical Sciences, Beijing, China

**Keywords:** TGF-β, Smad, β-catenin, diabetic nephropathy, Wnt

## Abstract

Diabetic nephropathy is a severe microvascular complication of diabetes. Qishen Yiqi dripping pill (QYDP) has been reported to be a renal protective drug. However, the mechanisms remain unclear. This study was performed to investigate the mechanisms. In this study, Sprague-Dawley rats were injected with streptozotocin to generate a diabetes model. Diabetic rats were administered 150 or 300 mg/kg/day QYDP. After 8 weeks of treatment, serum creatinine, serum blood urea nitrogen, and 24-h urinary albumin were measured. Kidney histological staining and immunostaining were analyzed. Then, the renal tissue was analyzed with a genome expression array. The results showed that QYDP treatment reduced serum creatinine, blood urea nitrogen, and 24-h urinary albumin and improved kidney histology and fibrosis. The gene array revealed that the expression of 189 genes was increased, and that of 127 genes was decreased in the high dosage QYDP group compared with the diabetic group. Pathway and gene ontology analyses showed that the differentially expressed genes were involved in the Wnt/β-catenin and transforming growth factor-β (TGF-β)/Smad2 signaling pathways. QYDP reduced the renal Wnt1, catenin β1, Tgfb1, and Smad2 gene expression and β-catenin, TGF-β, Smad2, collagen I, α-smooth muscle actin, and fibronectin protein expression in diabetic rats. Our results provide the first evidence that QYDP performs its renal-protective function by inhibiting the Wnt/β-catenin and TGF-β/Smad2 signaling pathways in diabetic rats.

## Introduction

In 2019, the International Diabetes Federation announced that diabetes affects approximately 463 million people worldwide. The number of affected individuals will reach 702 million by the year 2045 (Williams et al., 2020). Chronic tissue complications from diabetes worsen the health status of patients. Diabetic nephropathy (DN) is one of the most important microvascular complications. It is estimated that more than 20% of diabetic patients will develop chronic kidney disease (CKD) (Nelson et al., 1996). Nephropathy contributes to the development of a cardiovascular disease, resulting in increased all-cause mortality (Go et al., 2004).

The pathological characteristics of DN include the accumulation of extracellular matrix (ECM) in the glomerulus and tubules of the kidney, which leads to proteinuria and renal failure. Several pathways have been implicated in the underlying mechanisms of DN progression, such as oxidative stress (Baynes, 1991), inflammation (Mariappan, 2012), accumulation of advanced glycation end products (AGEs) (Brownlee et al., 1988), activation of protein kinase C (Koya et al., 1997), reactive oxygen species (Ha et al., 2008), and endoplasmic reticulum (ER) stress (Cybulsky et al., 2011).

Qishen Yiqi dripping pill (QYDP) is a traditional Chinese medicine compound that comprises Radix Astragali (*Astragalus penduliflorus* Lam.), redroot sage (*Salvia miltiorrhiza* Bunge), pseudoginseng (*Panax pseudoginseng* Wall.), and fragrant rosewood (*Dalbergia odorifera* T.C. Chen). QYDP is approved by the State Food and Drug Administration of China (state medical license no. Z20030139). A clinical trial showed that QYDP treatment reduced the urinary album excretion rate in diabetic patients (Yongbin Chen, 2011). QYDP intervention attenuated renal interstitial fibrosis in CKD model rats induced by unilateral ureteral obstruction surgery (Zhou et al., 2016). However, little is known about the mechanism of QYDP in the diabetic model.

The systematic principle of Chinese medicine raises the theory that traditional Chinese medicines have the integrated functions of all their constituents. Thus, we hypothesized that QYDP has multiple targets in the kidney and moderates the kidney function in diabetic rats. To identify these pathways, we used a genome-wide array approach to analyze gene expression changes in the kidney and pathway analysis to gain deep insight into the gene expression alternations.

## Materials and Methods

### Medicine

Qishen Yiqi dripping pill contains Radix Astragali (*A. penduliflorus* Lam., 62.24%), redroot sage (*S. miltiorrhiza* Bunge, 31.12%), pseudoginseng (*P. pseudoginseng* Wall., 6.22%), and fragrant rosewood (*D. odorifera* T.C. Chen, 0.42%). QYDP was provided by Tasly Pharmaceutical Group Co., Ltd. (Tianjin, China). All voucher specimens (no. QYDP19A-QYDP19D) were deposited at the Department of Endocrinology, Peking Union Medical College Hospital, Beijing, China. Detailed information about the four herbs is presented in Supplementary Table 1. The quality of the herbs and herbal extracts was consistent with the standards of Chinese Pharmacopoeia (2015). The four-component herbs, Radix Astragali (1,800 g), redroot sage (900 g), pseudoginseng (180 g), and fragrant rosewood (12 g), were soaked in 60% ethanol for 1 h and extracted twice by refluxing for 2 h. The condensed extracts were mixed with dextrin and sugar powder to produce QYDP.

### Ultra-Performance Liquid Chromatography Analysis of Qishen Yiqi Dripping Pill

Qishen Yiqi dripping pill powder (0.3 g) was dissolved in methanol and then filtered through a 0.22-μm filter membrane. QYDP was characterized using a Waters Acquity Ultra-Performance Liquid Chromatography (UPLC) (Waters Corp., Milford, MA, United States) with a symmetrical C18 column (100 × 2.1 mm i.d., particle size 1.7 μm, Waters Corp., Milford, MA, United States). The column was eluted at 30°C with a detection wavelength of 203 nm and an injection volume of 2 μl. The flow rate of the mobile phase of acetonitrile (A) and water (B) was set at 0.2 ml/min. Gradient separation was based on the following: 0–2 min, 15% A; 2–3 min, 15–18% A; 3–10 min, 18–20% A; 10–12 min, 20–25% A; 12–13.5 min, 25–34% A; 13.5–19 min, 34% A; 19–19.1 min, 34–90% A; 22–22.1 min, 90–15% A, 22.1–25 min, 15% A.

### Animal Treatments and Diet

A total of twenty four 5-week-old male Sprague-Dawley (SD) rats were purchased from the Institute of Laboratory Animal Sciences, Chinese Academy of Medical Sciences and Peking Union Medical College, Beijing, China and provided with a standard diet and water. The rats were kept under a 12-h light/12-h dark cycle and at 24°C. This study was conducted in strict accordance with the recommendations and with the approval of the Animal Care Committee of the Peking Union Medical Hospital Animal Ethics Committee (Project XHDW-2015-0051, 15 February 2015), and all efforts were made to minimize suffering. Diabetes was induced by injection of streptozotocin (Sigma-Aldrich, St. Louis, MO, United States) at a dose of 60 mg/kg body weight. The rats with fasted blood glucose levels > 11.1 mmol/L were considered diabetic. Diabetic rats were divided into three groups: a vehicle-treated group [diabetes mellitus (DM) group, *n* = 6], low dosage of Qishen Yiqi dripping pill group (*n* = 6), and high dosage of QishenYiqi dripping pill group (HQYDP group, *n* = 6). The typical human daily dose of QYDP is 1.5 g per 60 kg of body weight. Thus, according to the formula d_rat_ = (37 × d_human_)/6, the corresponding dose of QYDP for rats is 154.2 mg/kg per day. Previous reports show that there is no toxic reaction in rats treated with 4,000 mg/kg QYDP for 26 weeks (Wang et al., 2019). Therefore, the low dosage of Qishen Yiqi dripping pill and HQYDP groups were orally administered QYDP (Tasly Pharmaceutical Group Co., Ltd., Tianjin, China) at 150 and 300 mg/kg/day by gavage, respectively. The DM group and normal control (NC) group were given an equal volume of saline. All rats were anesthetized *via* intraperitoneal injection of sodium pentobarbital (150 mg/kg) and then killed at the eighth week after treatment. The kidneys were immediately collected.

### Blood Sample Analysis and Sample Preparation

After 6 h of fasting, blood was collected through the intraorbital retrobulbar plexus. For 24-h urine collection, the rats were housed in individual metabolic cages at the end of the 8-week treatment. Urine was centrifuged at 3,000 × *g* for 10 min at room temperature. Blood glucose, serum creatinine, blood urea nitrogen (BUN), and urine albumin levels were measured with a Beckman biochemical analyzer (Counter, AU5800, Germany).

### Histological Examination of the Kidney

Kidneys were fixed in formalin and then embedded in paraffin. Five-micrometer-thick sections were stained with periodic–acid Schiff (PAS) and Masson’s trichrome stain. Using PAS staining, the glomerular score of each rat was calculated as the arithmetic mean of 60 glomeruli (400× magnification) (el Nahas et al., 1987). The tubulointerstitial damage score (dilatation, atrophy, hyaline in the tubular lumen, visible detachment of tubular cells, interstitial infiltration of mononuclear cells, and interstitial fibrosis) was assessed as previously described (Piecha et al., 2008).

### RNA Extraction and Gene Array Analysis

Total RNA was extracted from the kidney cortex using a mirVana^TM^ RNA isolation kit (Ambion, Sãn Paulo, Brazil). Double-stranded complementary DNA (cDNA) was synthesized from RNA. Then, biotinylated cDNA was hybridized to an Affymetrix GeneChip Rat Gene 2.0 ST whole transcript-based array (Affymetrix Technologies, Santa Clara, CA, United States). Genes that had a *p-*value < 0.05 and a fold change > 1.5 were selected. The data obtained have been deposited in the National Center for Biotechnology Information Gene Expression Omnibus database (accession number GSE134072).

The Database for Annotation, Visualization, and Integrated Discovery web-based software tool was used to perform gene ontology (GO) enrichment analysis. In addition, pathway enrichment analysis based on the Kyoto Encyclopedia of Genes and Genomes (KEGG) database was used to identify significant pathways.

### Real-Time PCR Analysis

cDNA was synthesized using SuperScript II reverse transcriptase (Life Technologies, Carlsbad, CA, United States). Real-time PCR was performed using a Real-time PCR Mater Mix Kit (Applied Biosystems, Foster City, CA, United States) and ABI SYBR Mix (Applied Biosystems, Foster City, CA, United States). The specific primers are listed in Table 1. Data were analyzed using the ΔΔCt method with glyceraldehyde 3-phosphate dehydrogenase as the constitutive marker.

**TABLE 1:** Oligonucleotide sequences for qPCR analysis.

Gene symbol	Gene bank ID	Forward primer	Reverse primer	Product size (bp)
Wnt1	NM_001105714	TCTTCTCGGGA GACCCCTTT	ATACCACAGGG ACAGCAACG	124
Ctnnb1	NM_053357	ATCATTCTGGCC AGTGGTGG	GACAGCACCTTC AGCACTCT	104
Tgfb1	NM_021578	AGGGCTACCAT GCCAACTTC	CCACGTAGTAG ACGATGGGC	168
Smad2	NM_001277450	GCCGCCCGAA GGGTAGAT	TTCTGTTCTCC ACCACCTGC	164

*Wnt1, Wnt family member 1; Ctnnb1, catenin beta 1; Tgfb1, transforming growth factor beta 1.*

### Immunohistochemistry for Transforming Growth Factor-β, β-Catenin, and Smad2 in the Kidney

Five-micron-thick renal sections were deparaffinized, rehydrated, and immersed in phosphate-buffered saline. Then, the sections were stained with anti-TGF-β (1:100, Santa Cruz Biotechnology, Dallas, TX, United States), anti-β-catenin (1:100, Santa Cruz Biotechnology, Dallas, TX, United States), and anti-Smad2 (1:100, Santa Cruz Biotechnology, Dallas, TX, United States) antibodies at 4°C overnight. Tissue sections were then incubated with a horseradish peroxidase-conjugated secondary antibody (1:2,000, Santa Cruz Biotechnology, Dallas, TX, United States) for 1 h at room temperature. Immuno-labeling was visualized with 0.05% diaminobenzidine. A digital microscope (Nikon, Tokyo, Japan) was used to analyze sections at 400 × magnification to identify positive cells.

### Western Blot Analysis

Kidneys were homogenized in radioimmunoprecipitation assay buffer (Millipore, Bedford, MA, United States) to obtain total proteins. Total protein (30 μg) was loaded in 10% sodium dodecyl sulfate-polyacrylamide gels and transferred to polyvinylidene fluoride membranes (Bio-Rad, Hercules, CA, United States). Then, the membranes were blocked in Tris-buffered saline with skim milk for 1 h, followed by overnight incubation at 4°C with rabbit anti-TGF-β (1:1,000, Abcam, Cambridge, United Kingdom), rabbit anti-β-catenin (1:1,000, Abcam, Cambridge, United Kingdom), rabbit anti-Smad2 (1:1,000, Abcam, Cambridge, United Kingdom), rabbit anti-collagen I (1:1,000, Abcam, Cambridge, United Kingdom), rabbit anti-α-smooth muscle actin (α-SMA, 1:1,000, Abcam, Cambridge, United Kingdom), or rabbit anti-fibronectin (FN, 1:1,000, Abcam, Cambridge, United Kingdom) antibody. After washing, the membranes were incubated with horseradish peroxidase-conjugated secondary antibody (1:3,000, Santa Cruz Biotechnology, Santa Cruz, CA, United States) for 2 h at room temperature. After another wash, membranes were developed using an enhanced chemiluminescence (Cell Signaling Technology, Danvers, MA, United States) assay. Bound proteins were scanned with an Epson V300 scanning system (Epson, Suwa, Japan). The density of protein bands was quantified with AlphaEaseFC software (Alpha Innotech, San Leandro, CA, United States). The housekeeping protein β-actin (1:3,000, Abcam, Cambridge, United Kingdom) was used for normalization.

### Statistical Analysis

Data are shown as the mean ± SD. Statistical analyses were calculated with two-way ANOVA followed by Tukey’s *post hoc* test among the four groups. The Student’s *t*-test was used to analyze differences between different groups. GraphPad Prism 6 (GraphPad Software Inc., CA, United States) was used for data analysis. *P* < 0.05 was considered to indicate significance.

## Results

### Ultra-Performance Liquid Chromatography Analysis of Qishen Yiqi Dripping Pill

**FIGURE 1:**
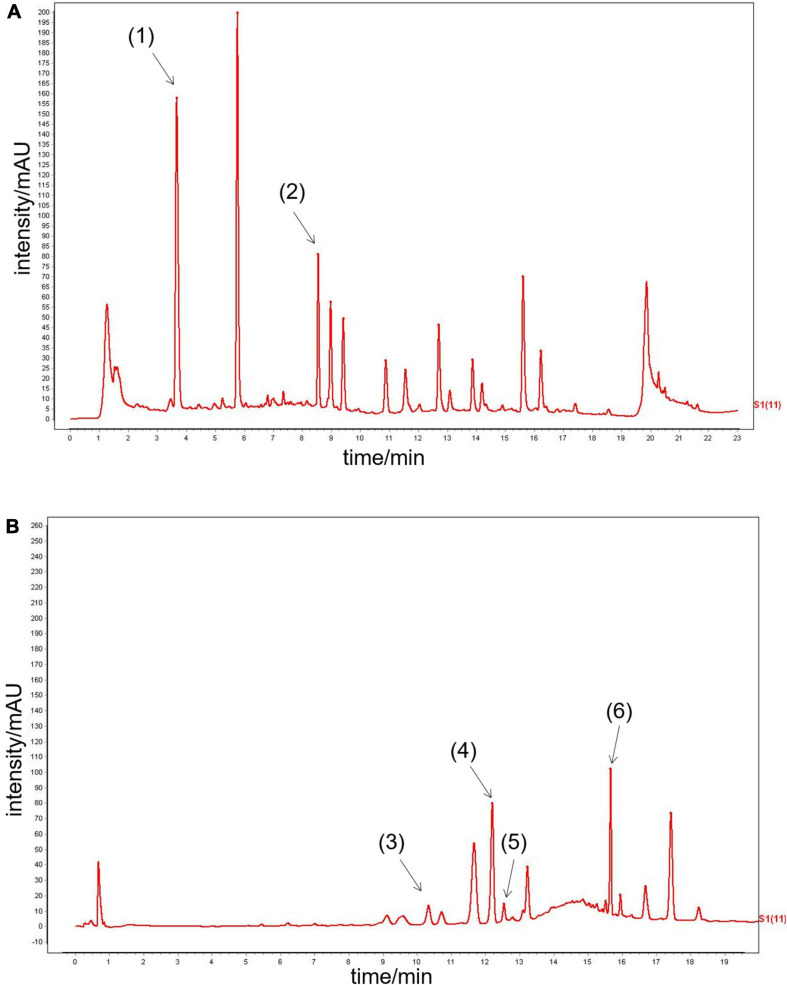
UPLC analysis of QYDP. **(A)** (1) salvianic acid, (2) calycosin glycoside, **(B)** (3) notoginseng R1, (4) Ginsenoside Rg1, (5) Ginsenoside Re, and (6) Ginsenoside Rb1.

Six main QYDP components were confirmed by UPLC analysis. The UV detector for UPLC analysis was set to 203 nm according to the standard maximum absorption rate. The UPLC analysis of QYDP is presented in Figures 1A,B. The six main QYDP constituents are (1) salvianic acid (3.942 mg/g), (2) calycosin glycoside (0.2656 mg/g), (3) notoginseng R1 (0.618 mg/g), (4) Ginsenoside Rg1 (2.204 mg/g), (5) Ginsenoside Re (0.4484 mg/g), and (6) Ginsenoside Rb1 (1.9 mg/g).

### Effect of Qishen Yiqi Dripping Pill on Body Weight and Fasting Blood Glucose

**FIGURE 2:**
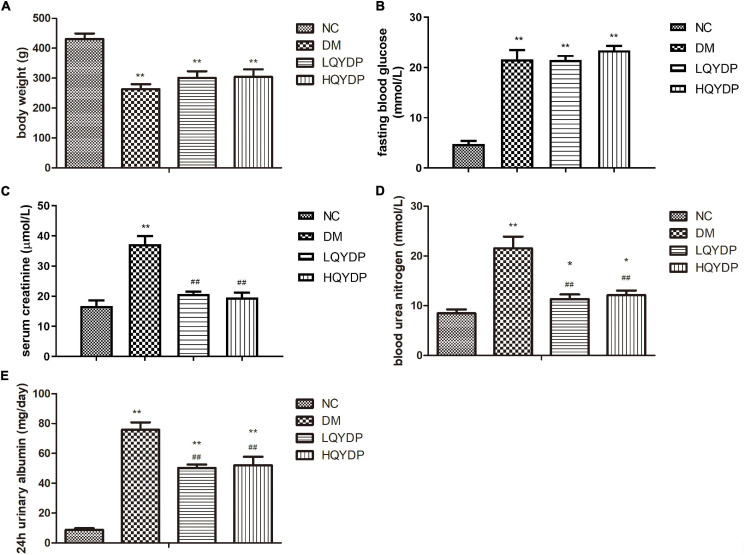
Effect of QYDP on **(A)** body weight, **(B)** fasting blood glucose, **(C)** serum creatinine, **(D)** blood urea nitrogen, and **(E)** 24-h urinary albumin in rats. Values are mean ± SD (*n* = 6); **p < 0.01, *p < 0.05 compared with NC group; ##*p* < 0.01 compared with DM group. QYDP, Qishen Yiqi dripping pill; NC, normal control; DM, diabetes mellitus; LQYDP, low dose of Qishen Yiqi dripping pill; HQYDP, high dose of Qishen Yiqi dripping pill.

The DM group showed decreased body weight compared with that of the NC group (*p* < 0.01, Figure 2A). QYDP did not change the body weight of diabetic rats (*p* > 0.05, Figure 2A). The DM group had higher fasting blood glucose (*p* < 0.01, Figure 2B) than the control group. QYDP did not reduce fasting blood glucose in diabetic rats (*p* > 0.05, Figure 2B).

### Effect of Qishen Yiqi Dripping Pill on Renal Function Parameters

Serum creatinine, BUN, and 24-h urinary albumin levels were significantly increased in the DM group (*p* < 0.01, Figures 2C–E). QYDP treatment decreased serum creatinine, BUN, and 24-h urinary albumin levels (*p* < 0.01, Figures 2C–E). These results suggest that QYDP moderated renal function in diabetic rats.

### Effect of Qishen Yiqi Dripping Pill Treatment on Histopathological Changes in Renal Tissue

**FIGURE 3:**
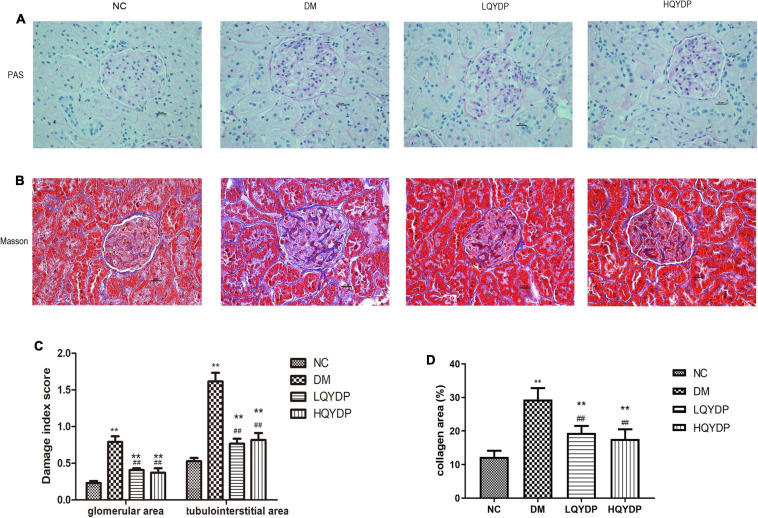
Effect of QYDP on histopathological changes in renal tissues of diabetic rats. **(A)** PAS stained kidney sections taken from rats in each group (400 × magnification), **(B)** Masson-stained kidney sections taken from rats in each group (400 × magnification), **(C)** glomerular and tubulointerstitial damage index score analysis with PAS staining. **(D)** Quantitative assessments of Masson-positive area (%) with Masson staining. Values are mean ± SD (*n* = 6); **p < 0.01 compared with NC group; ##*p* < 0.01 compared with DM group. QYDP, Qishen Yiqi dripping pill; NC, normal control; DM, diabetes mellitus; LQYDP, low dose of Qishen Yiqi dripping pill; HQYDP, high dose of Qishen Yiqi dripping pill.

With PAS staining, both a higher glomerular lesion score and higher tubulointerstitial lesion score were observed in diabetic rats compared with normal rats (*p* < 0.01, Figures 3A,C). Glomerular hypertrophy and tubulointerstitial changes were mostly prevented by QYDP treatment (*p* < 0.01, Figures 3A,C). Further examination of Masson’s-stained renal tissue sections showed that the diabetic rats presented more collagen fibers in the glomerular mesangium and basement membrane (*p* < 0.01, Figures 3B,D). QYDP treatment significantly attenuated collagen deposition (*p* < 0.01, Figures 3B,D).

### Gene Array, Pathway, Gene Ontology, and Network Analysis Results in the High Dosage of QishenYiqi Dripping Pill Group vs. the Diabetes Mellitus Group

**FIGURE 4:**
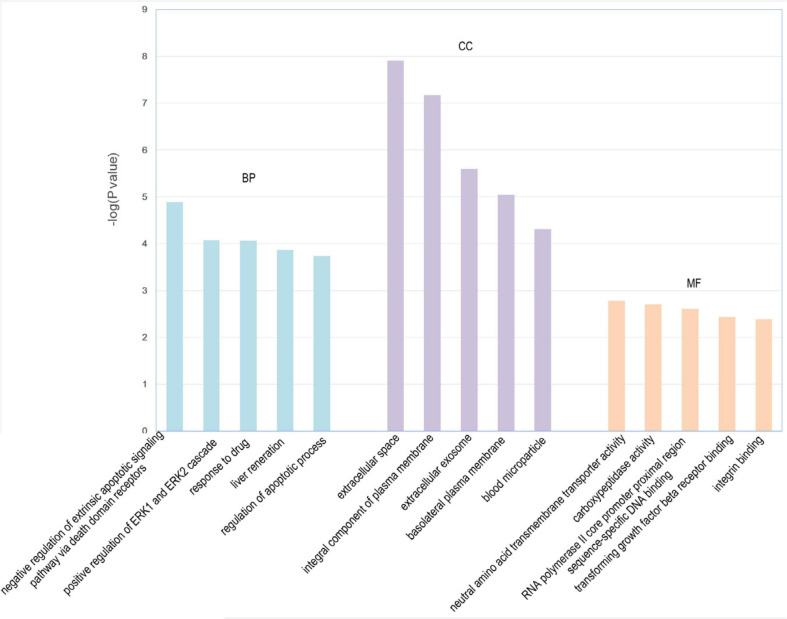
Enriched GO terms associated with differentially expressed genes in HQYDP group vs. DM group. Top five terms in biological process (BP), cellular component (CC), and molecular function (MF).

**FIGURE 5:**
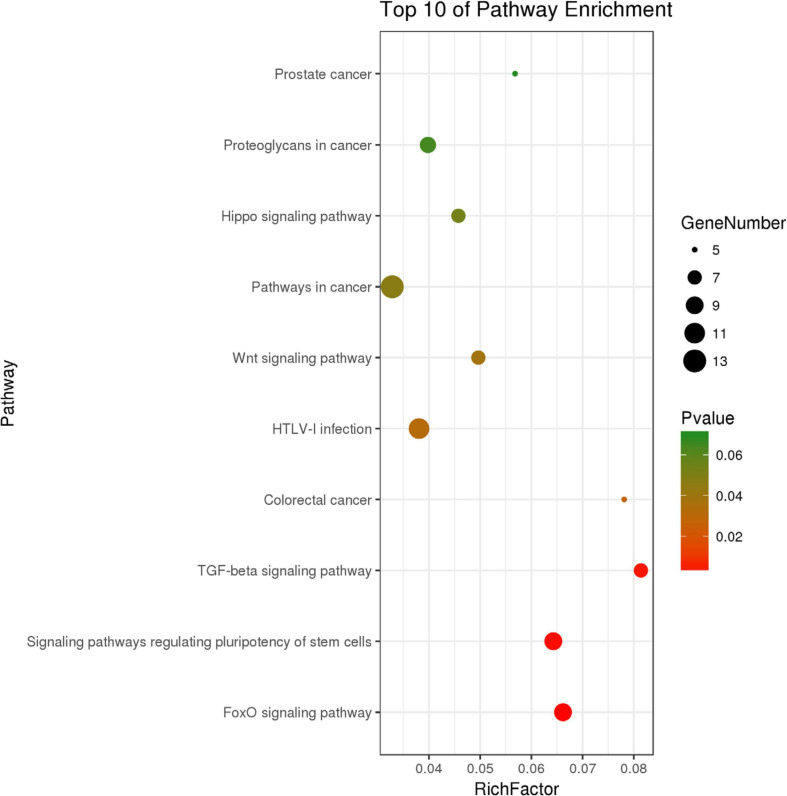
Top 10 KEGG pathway enrichment point diagram in HQYDP group vs. DM group. Vertical axis represents pathway name, horizontal axis represents Rich factor, size of dot indicates number of genes expressed in pathway, and color of dot corresponds to different *p* values.

We identified 316 significantly differentially expressed genes, including 189 upregulated genes and 127 downregulated genes in the HQYDP group, compared with the DM group (fold change > 1.5, *p* < 0.05). To systematically identify biological connections among the differentially expressed genes and to identify pathways associated with the effect of QYDP on the kidney, we performed GO and KEGG pathway analyses. Figure 4 and Table 2 show the top five terms in three categories, biological processes, cellular components, and molecular functions. Among the biological process ontology results, the major GO terms affected by QYDP were negative regulation of extrinsic apoptotic signaling pathway *via* death domain receptors, positive regulation of ERK1 and ERK2 cascade, response to a drug, liver regeneration, and regulation of the apoptotic process. Figure 5 and Table 3 show the top 10 pathways identified *via* KEGG pathway analysis affected by QYDP, including the FoxO signaling pathway, signaling pathways regulating pluripotency of stem cells, TGF-β signaling pathway, colorectal cancer, Human T- cell leukemia virus, type 1 infection, Wnt signaling pathway, pathways in cancer, Hippo signaling pathway, proteoglycans in cancer, and prostate cancer.

**TABLE 2:** Top 5 enriched GO terms in each catalog associated with differentially expressed genes in HQYDP group vs. DM group.

term ID	term name	count	*p* value	fold enrichment	involved genes	catalog
GO:1902042	negative regulation of extrinsic apoptotic signaling pathway via death domain receptors	6	1.29 × 10^–5^	18.828	ICAM1, FGG, FGA, SFRP2, FGB, HMOX1	biological processes
GO:0070374	positive regulation of ERK1 and ERK2 cascade	12	8.42 × 10^–5^	4.454	ICAM1, FGG, FGA, C1QTNF3, FGB, SEMA7A, P2RY1, PYCARD, TREM2, GPNMB, SLAMF1, TGFB1	biological processes
GO:0042493	response to drug	21	8.64 × 10^–5^	2.746	ICAM1, SLC8A1, MAT2A, LGALS1, IL1RN, GGH, CFTR, ABCA1, SLCO1A6, TGFB1, CTNNB1, CDKN1A, TNFRSF11B, ACE, HTR1B, PLIN2, SFRP2, ABCB1B, TGIF1, FAS, MYC	biological processes
GO:0097421	liver regeneration	7	1.36 × 10^–4^	8.786	FGA, HMOX1, CLDN1, RGN, FAS, MYC, TGFB1	biological processes
GO:0042981	regulation of apoptotic process	11	1.84 × 10^–4^	4.467	TNFRSF1B, TNFRSF11B, BMP1, SFRP2, PYCARD, CIDEA, APAF1, FAS, GDF15, MYC, CTNNB1	biological processes
GO:0005615	extracellular space	47	1.24 × 10^–8^	2.496	GC, LTBP4, BTC, UMOD, TGFB1, LIF, WNT1, FGG, TNFRSF11B, WNT4, ACE, FGA, C1QTNF3, FGB, SEMA7A, PPP1R1A, VNN1, TFF3, SERPINB12, FAS, EGF, GCNT1, ANGPTL4, SPP1, SELP, ICAM1, BMP1, PLA2G15, LOC360919, C4B, LGALS1, IL1RN, KNG1L1, AXL, GGH, CPXM2, HILPDA, MFGE8, AFM, DKK1, SFRP2, LIPG, ACE2, ANXA13, IGFBP1, GDF15, PON3	cellular components
GO:0005887	integral component of plasma membrane	37	6.76 × 10^–8^	2.745	LOC361914, CLDN4, CADM2, SLC15A2, TSPAN4, KCNJ10, ABCA1, ATP12A, KCNJ13, INSRR, TNFRSF1B, SLCO1A1, TNFRSF11B, HTR1B, SLC39A8, FAS, GPNMB, SLC22A2, ICAM1, SLC8A1, SLC6A13, SLC22A22, NPR2, SLC10A5, SLC7A13, SLCO1A6, SLC7A12, SLC26A4, SEMA6A, FOLH1, SLC16A4, SLC16A7, GRM8, SLC26A7, CLDN1, SLC13A1, STEAP1	cellular components
GO:0070062	extracellular exosome	67	2.55 × 10^–6^	1.770	PVR, GDA, SLC15A2, LTBP4, CTNNB1, SLC1A4, PRKAR2A, TUBB6, VNN1, RHOB, FAS, ATP6V0D2, SLC22A2, ICAM1, HIST1H1B, C4B, CFTR, MFGE8, BASP1, VAT1, SLC26A4, FOLH1, PSCA, PRPS2, MYO5A, GC, ACADSB, UMOD, ATP6V1B1, FGG, ACE, FGA, HNMT, C1QTNF3, FGB, TFF3, NDRG1, SERPINB12, EGF, SPP1, NAT8, DNM3, PLA2G15, CPNE4, HIST1H2BF, LGALS1, IL1RN, SLC6A13, KNG1L1, GGH, AXL, NID2, CRYZ, TPMT, CPVL, SLAMF1, AFM, PPIC, ACE2, MEP1A, ANXA13, HIST1H2AH, HIST1H2AK, PAPPA2, APAF1, GDF15, PON3	cellular components
GO:0016323	basolateral plasma membrane	14	9.07 × 10^–6^	4.727	SLC8A1, CASR, NKD2, SLC22A22, UMOD, CFTR, KCNJ10, ATP6V1B1, ATP12A, CTNNB1, SLCO1A1, SLC26A7, P2RY1, SLC22A2	cellular components
GO:0072562	blood microparticle	10	4.90 × 10^–5^	5.923	GC, FGG, AFM, FGA, LOC691828, C4B, FGB, KNG1L1, TGFB1, ANGPTL4	cellular components
GO:0015175	neutral amino acid transmembrane transporter activity	4	0.0017	16.453	SLC1A4, LOC361914, SLC7A13, SLC7A12	molecular function
GO:0004180	carboxypeptidase activity	4	0.0020	15.485	FOLH1, ACE, ACE2, CPXM2	molecular function
GO:0000978	RNA polymerase II core promoter proximal region sequence-specific DNA binding	14	0.0024	2.663	AR, ELF3, SPI1, SMAD3, SMAD2, MYBL1, FOXP2, GCM1, NR1D1, OVOL1, TEF, TGIF1, POU3F1, MYC	molecular function
GO:0005160	transforming growth factor beta receptor binding	5	0.0037	7.835	BMP1, SMAD3, SMAD2, GDF15, TGFB1	molecular function
GO:0005178	integrin binding	7	0.0041	4.607	ICAM1, CASR, SEMA7A, TSPAN4, MFGE8, GPNMB, SYK	molecular function

**TABLE 3:** Top 10 enriched KEGG pathways associated with differentially expressed genes in HQYDP group vs. DM group.

pathway ID	pathway term	count	fold enrichment	*p* value	involved genes
rno04068	FoxO signaling pathway	9	3.716	0.003	G6PC, SGK1, CDKN1A, S1PR1, PLK2, SMAD3, SMAD2, EGF, TGFB1
rno04550	Signaling pathways regulating pluripotency of stem cells	9	3.610	0.003	LIF, WNT1, WNT4, SMAD3, SMAD2, ID4, ID3, MYC, CTNNB1
rno04350	TGF-beta signaling pathway	7	4.571	0.004	TGIF1, SMAD3, SMAD2, ID4, ID3, MYC, TGFB1
rno05210	Colorectal cancer	5	4.387	0.026	SMAD3, SMAD2, MYC, TGFB1, CTNNB1
rno05166	HTLV-I infection	11	2.137	0.032	ICAM1, WNT1, CDKN1A, WNT4, SPI1, SMAD3, SMAD2, MYC, TGFB1, CTNNB1, TP53INP1
rno04310	Wnt signaling pathway	7	2.788	0.039	WNT1, WNT4, NKD2, DKK1, SFRP2, MYC, CTNNB1
rno05200	Pathways in cancer	13	1.843	0.047	WNT1, CDKN1A, AR, WNT4, SPI1, GNG13, SMAD3, SMAD2, FAS, EGF, MYC, TGFB1, CTNNB1
rno04390	Hippo signaling pathway	7	2.569	0.054	WNT1, WNT4, SMAD3, SMAD2, MYC, TGFB1, CTNNB1
rno05205	Proteoglycans in cancer	8	2.235	0.065	WNT1, CDKN1A, WNT4, HPSE2, FAS, MYC, TGFB1, CTNNB1
rno05215	Prostate cancer	5	3.190	0.070	CDKN1A, AR, EGF, CTNNB1, INSRR

**FIGURE 6:**
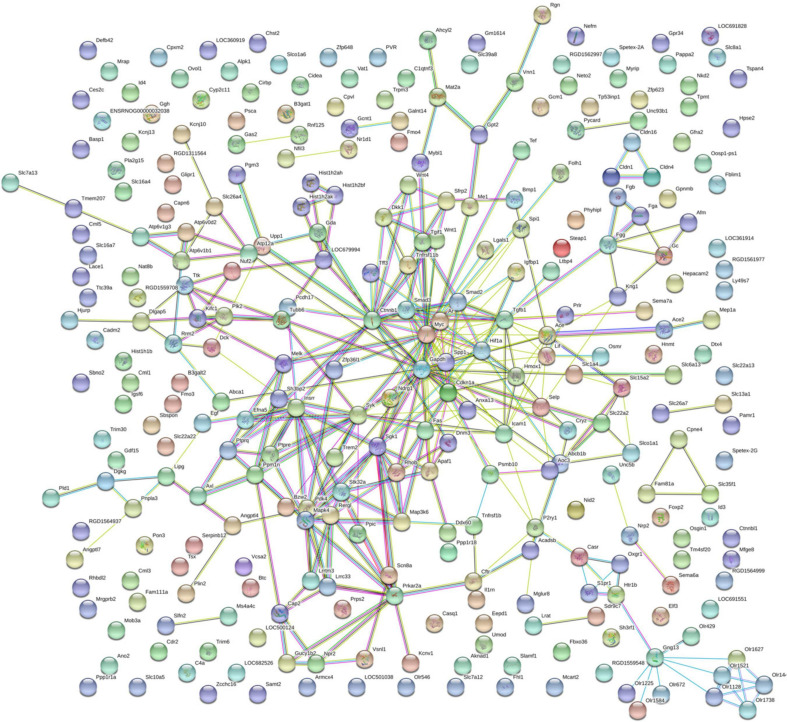
Protein–protein interaction network in HQYDP group compared with DM group. Nodes stand for differentially expressed genes in HQYDP group compared with DM group. Lines represent interactions between two proteins.

By using String online software, the 316 differentially expressed genes were mapped in a network. Two hundred ninety-six nodes with 350 joint-edges were featured in this map (Figure 6). Fifteen nodes with no less than 10 joint-edges were considered important functional molecules in QYDP-treated kidneys because they accounted for 66.8% of the function of all genes. Among these 15 nodes, glyceraldehyde-3-phosphate dehydrogenase, myelocytomatosis oncogene, catenin β1 (Ctnnb1), transforming growth factor β1 (Tgfb1), serum glucocorticoid regulated kinase 1, spleen tyrosine kinase, cyclin-dependent kinase inhibitor 1 A, mitogen-activated protein kinase 4, angiotensin I converting enzyme, and intercellular adhesion molecule 1 were ranked in the top 10.

### Real-Time PCR Validation Results

**FIGURE 7:**
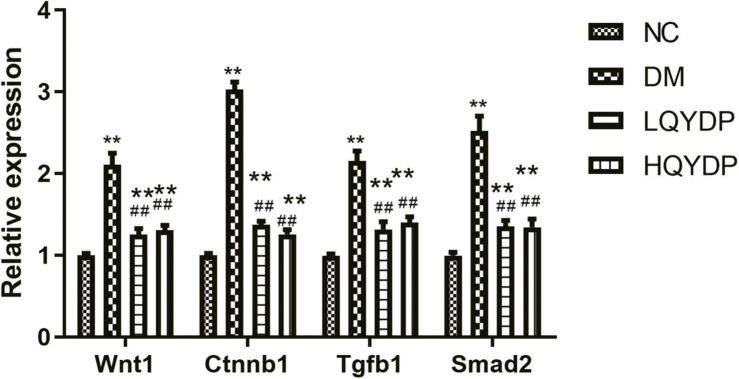
Effect of QYDP on Wnt1, Ctnnb1, Tgfb1, and Smad2 expression determined by qPCR. Values are mean ± SD (*n* = 6); **p < 0.01 compared with NC group; ##*p* < 0.01 compared with DM group. QYDP, Qishen Yiqi dripping pill; NC, normal control; DM, diabetes mellitus; LQYDP, low dose of QishenYiqi dripping pill; HQYDP, high dose of QishenYiqi dripping pill; Wnt1, Wnt family member 1; Ctnnb1, catenin beta 1; Tgfb1, transforming growth factor beta 1.

Because the TGF-β signaling pathway and Wnt signaling pathway were among the top-ranked KEGG pathways associated with differentially expressed genes in the HQYDP group vs. the DM group, we selected several genes in these two pathways to assay expression among the four different groups using quantitative PCR. We found that diabetic rats had significantly increased Wnt family member 1 (Wnt1), Ctnnb1, Tgfb1, and Smad2 expression in the kidney (*p* < 0.01, Figure 7) compared with that in the control group. QYDP treatment reduced Wnt1, Ctnnb1, Tgfb1, and Smad2 expression in diabetic kidneys (*p* < 0.01, Figure 7). These results were consistent with the microarray results.

### Effect of Qishen Yiqi Dripping Pill on Transforming Growth Factor-β, β-Catenin, and Smad2 Expression in the Kidney Determined by Immunohistochemistry Staining

**FIGURE 8:**
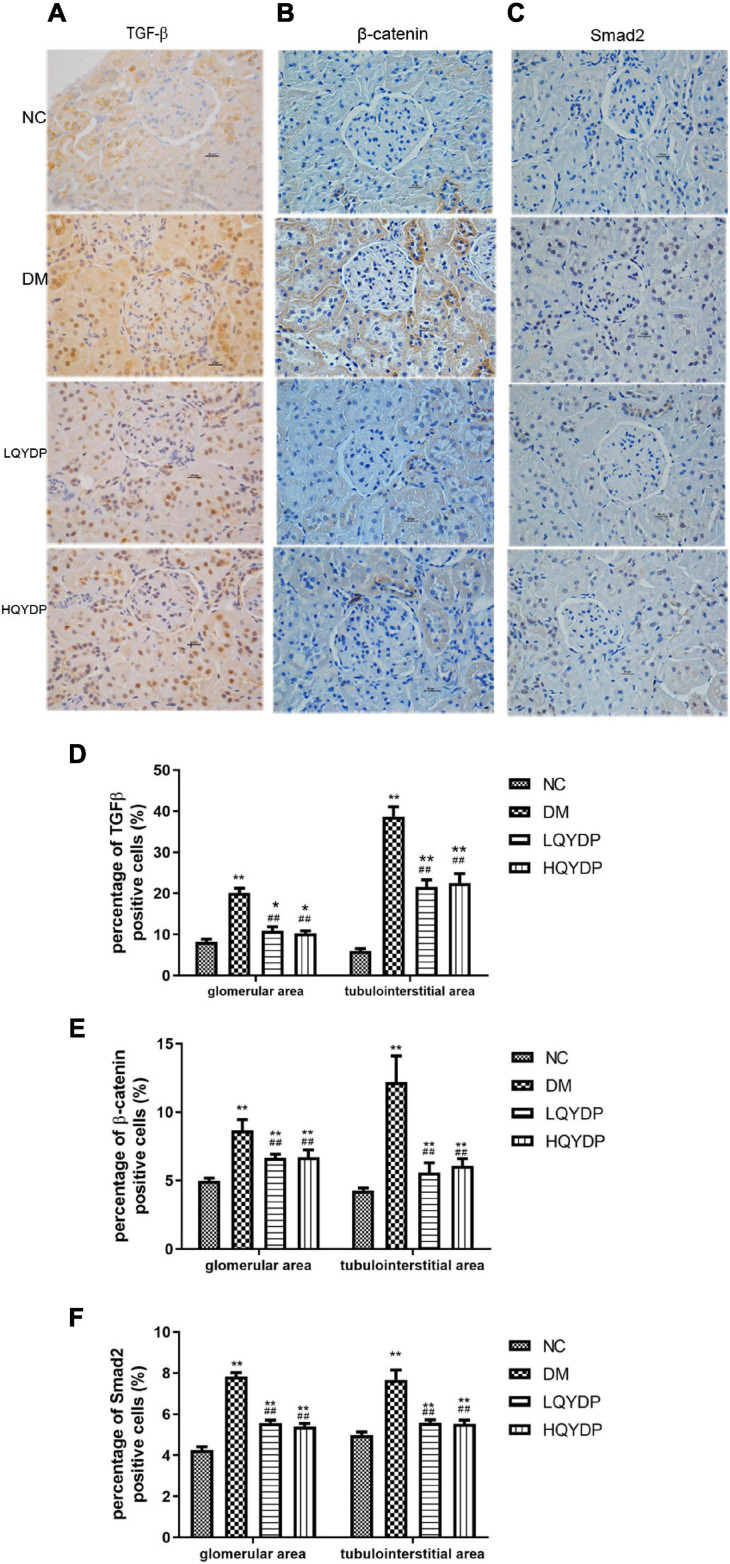
Effect of QYDP on TGF-β, β-catenin, and Smad2 expression shown by immunohistochemistry staining. **(A)** immunostaining for TGF-β, **(B)** immunostaining for β-catenin, **(C)** immunostaining for Smad2, **(D)** percentage (%) of TGF-β (+) in kidneys, **(E)** percentage (%) of β-catenin (+) cells in kidneys, and **(F)** percentage (%) of Smad2 (+) cells in kidneys. Values are mean ± SD (*n* = 6); *p < 0.05, **p < 0.01 compared with normal group; ##*p* < 0.01 compared with DM group. NC, normal control; DM, diabetes mellitus; LQYDP, low dose of Qishen Yiqi dripping pill; HQYDP, high dose of Qishen Yiqi dripping pill.

Immunohistochemistry analyses showed that in the DM group, TGF-β, β-catenin, and Smad2 immunoreactivity was higher than in the NC group in the glomeruli, tubuli, and interstitial areas (*p* < 0.01, Figure 8). QYDP treatment inhibited TGF-β, β-catenin, and Smad2 levels in the glomerular and tubulointerstitial areas of diabetic kidneys (*p* < 0.01, Figure 8).

### Effect of Qishen Yiqi Dripping Pill on the Protein Levels of Transforming Growth Factor-β, β-Catenin, Smad2 and Fibrotic Markers in the Kidney

**FIGURE 9:**
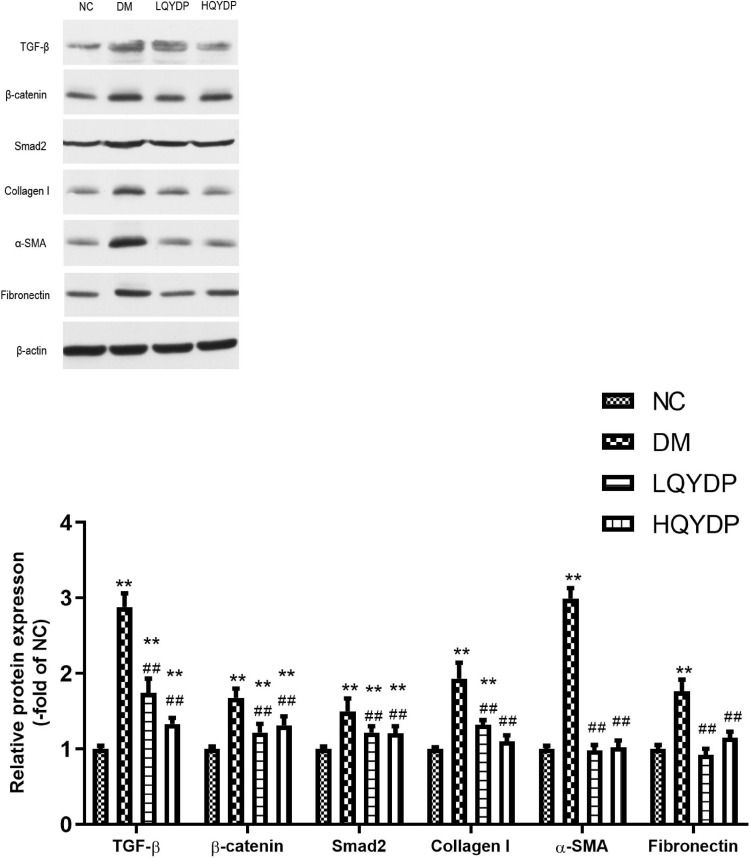
Effect of QYDP on TGF-β, β-catenin, Smad2, collagen I, α-SMA, and fibronectin protein levels in kidneys. Values are mean ± SD (*n* = 6); **p < 0.01 compared with NC group; ##*p* < 0.01 compared with DM group. QYDP, Qishen Yiqi dripping pill; NC, normal control; DM, diabetes mellitus; LQYDP, low dose of Qishen Yiqi dripping pill; HQYDP, high dose of Qishen Yiqi dripping pill.

Similar to the immunohistochemical analyses, an increase in TGF-β, β-catenin, and Smad2 was observed in diabetic rat kidneys (*p* < 0.01, Figure 9). Treatment with QYDP significantly decreased TGF-β, β-catenin, and Smad2 expression (*p* < 0.01, Figure 9). The expression levels of the fibrotic markers, collagen I, α-SMA, and FN were upregulated in diabetic rat kidneys (p < 0.01, Figure 9). QYDP treatment reversed the increases of these fibrotic markers (p ( 0.01, Figure 9).

## Discussion

In this study, our data show that both the low and high QYDP dosage reduced serum creatinine, BUN, and 24-h urinary albumin and moderated kidney hypertrophy and renal histology in diabetic rats. No significant dosage-dependent effect was observed. The possible reason might be that the high dosage exceeds the maximum efficacy. Furthermore, we observed a significant downregulation of collagen I, α-SMA, and FN in QYDP-treated diabetic rats. Overall, QYDP moderated kidney function and renal fibrosis in diabetic rats. QYDP includes Radix Astragali (*A. penduliflorus* Lam.), redroot sage (*S. miltiorrhiza* Bunge), pseudoginseng (*P. pseudoginseng* Wall.), and fragrant rosewood (*D. odorifera* T.C. Chen). Astragalosides, which is an active ingredient of Radix Astragali, have a potent antioxidative effect and inhibit high glucose-induced mesangial cell proliferation *in vitro* (Sun et al., 2014; Chen et al., 2016). Radix Astragali significantly reduces oxidative activity in diabetic rat kidneys (Chen et al., 2016). Two major isoflavonoids in Radix Astragali have inhibitory effects on AGE-induced endothelial cell apoptosis (Tang et al., 2011). Redroot sage extracts have a renoprotection effect in streptozotocin-induced diabetic rats (Yin et al., 2014), ameliorated renal function, and reduced TGF-β1 (Liu et al., 2005) and collagen IV (Lee et al., 2011).

Without reducing blood glucose, QYDP can moderate renal function in diabetic rats. This indicates that QYDP has direct beneficial effects on the kidney rather than indirect effects through moderating hyperglycemia. We further performed microarray gene expression profile analysis to determine the mechanism underlying the effect of QYDP on the kidneys of diabetic rats. Then, pathway analysis of the genes that were differentially expressed in the HQYDP-treated group compared with the DM group was performed. From this analysis, we found that the Wnt signaling pathway was in the top 10 pathways. The Wnt signaling pathway diversifies into two branches: the canonical Wnt pathway (the β-catenin pathway) and the non-canonical Wnt pathway. In this study, we found that QYDP reduced Wnt1 and Ctnnb1 expression in diabetic rats. Additionally, immunostaining analysis and western blotting showed that QYDP reduced β-catenin protein expression. In gene interaction analysis, Ctnnb1 was centrally located among all the differentially expressed genes in the HQYDP-treated group compared with those in the DM group. In CKD rats, QYDP also inhibited β-catenin expression (Zhou et al., 2016). The Wnt/β-catenin pathway is involved in cellular growth and differentiation in DN (Xiao et al., 2013). In kidneys of both type 1 and 2 diabetic models, the Wnt pathway is activated abnormally (Zhou et al., 2012). This activation of Wnt/β-catenin contributes to proteinuria (Kato et al., 2011), podocyte dysfunction (Dai et al., 2009), glomerulosclerosis, and renal interstitial fibrosis (He et al., 2009). *In vivo* and *in vitro*, overexpression of either Wnt1 or stabilized β-catenin leads to podocyte dysfunction (Dai et al., 2009). Blockade or knockout of Wnt/β-catenin protects against the development of podocyte lesions and albuminuria (Dai et al., 2009). Thus, QYDP treatment may alleviate kidney dysfunction by inhibiting the Wnt/β-catenin pathway.

In addition, pathway analysis showed that the TGF-β signaling pathway ranked third among all pathways affected by QYDP in the kidneys. Immunostaining analysis and western blot analysis showed that QYDP reduced TGF-β expression. TGF-β is an essential mediator that stimulates glomerular ECM formation in DN (Yamamoto et al., 1994). Hyperglycemia leads to TGF-β activation. Chronic inhibition of TGF-β using neutralizing monoclonal antibody in db/db mice prevents glomerulosclerosis and renal dysfunction (Ziyadeh et al., 2000). Zhou et al. (2016) found that QYDP inhibits TGF-β1-induced β-catenin upregulation in the cytoplasm but does not affect Smad2 and Smad3 phosphorylation and Smad4 or Smad7 expression in normal kidney proximal tubular (NRK52E) cells. However, in our study, gene interaction analysis showed that Smad2, Smad3, and Tgfb1 were in the central position among all the differentially expressed genes in the HQYDP-treated group compared with the DM group. QYDP reduced Tgfb1 and Smad2 expression and inhibited TGF-β and Smad2 protein expression in diabetic kidneys. The reason for the different results in NRK52E cells and diabetic rats might be the different circumstances *in vivo* and *in vitro*. Unlike diabetic rats, NRK52E cells were used by Zhou et al. (2016). The Smad signaling pathway plays an important role in the TGF-β1-stimulated accumulation of ECM. TGF-β1/Smad signaling is a critical pathway for the development of renal fibrosis. TGF-β1 activates Smad2 and Smad3 phosphorylation, and then, phospho-Smad2/3 binds to Smad4 to form hetero-oligomeric complexes (Meng et al., 2015). These complexes translocate to the nucleus to regulate the transcription factors of other genes related to kidney fibrosis (Massague and Chen, 2000). Hence, QYDP might inhibit TGF-β1/Smad signaling to improve kidney function in diabetic rats.

## Conclusion

**FIGURE 10:**
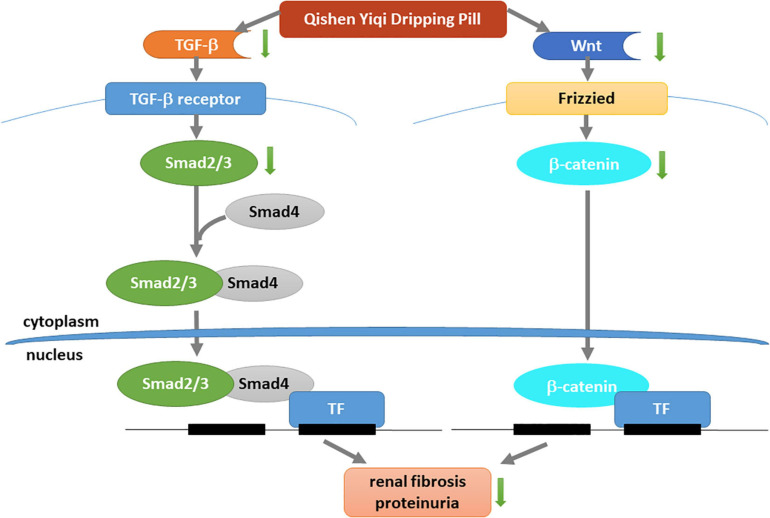
Mechanism underlying renal-protective effect of QYDP. QYDP inhibits Wnt/β-catenin signaling pathway and TGF-β/Smad signaling pathway to reduce renal fibrosis and proteinuria in diabetic rats.

In summary, this study reveals that QYDP significantly attenuates kidney function and renal fibrosis. Our study is the first to show that QYDP moderates kidney function by inhibiting the Wnt/β-catenin pathway and TGF-β/Smad signaling in diabetic rats (Figure 10). These results provide a basis for the treatment of DN patients in the future. Further *in vitro* research is still required to elucidate the mechanistic details of QYDP protection against DN.

## Data Availability Statement

The datasets presented in this study can be found in online repositories. The names of the repository/repositories and accession number(s) can be found in the article/Supplementary Material.

## Ethics Statement

The animal study was reviewed and approved by this study was conducted in strict accordance with the recommendations and the approval of the Animal Care Committee of the Peking Union Medical Hospital Animal Ethics Committee (Project XHDW-2015-0051, 15 February 2015), and all efforts were made to minimize suffering.

## Author Contributions

XX designed the experiments and contributed reagents and materials. QZ, JZ, TW, and XW conducted the experiments. MY, ML, and FP analyzed the data. QZ wrote the manuscript. All authors have read and approved the article.

## Conflict of Interest

The authors declare that the research was conducted in the absence of any commercial or financial relationships that could be construed as a potential conflict of interest.
