# Supplementary material for: Qishen Yiqi Dripping Pill Protects Against Diabetic Nephropathy by Inhibiting the Wnt/β-Catenin and Transforming Growth Factor-β/Smad Signaling Pathways in Rats
Source: Front Physiol. 2021 Feb 19;11:613324. doi: 10.3389/fphys.2020.613324 (PMC7933526; doi:10.3389/fphys.2020.613324)
Supplement: Supplementary file 1 [file Table_1.doc]

**Supplementary Table 1** Detailed information of the crude drugs composed in QYDP.

| No. | Herb name | Batch number | Manufacture | Voucher specimen |
| --- | --- | --- | --- | --- |
| QYDP19A | Radix astragali | 2003001 | Gansu Zhongtian Pharmaceutical Co., Ltd | 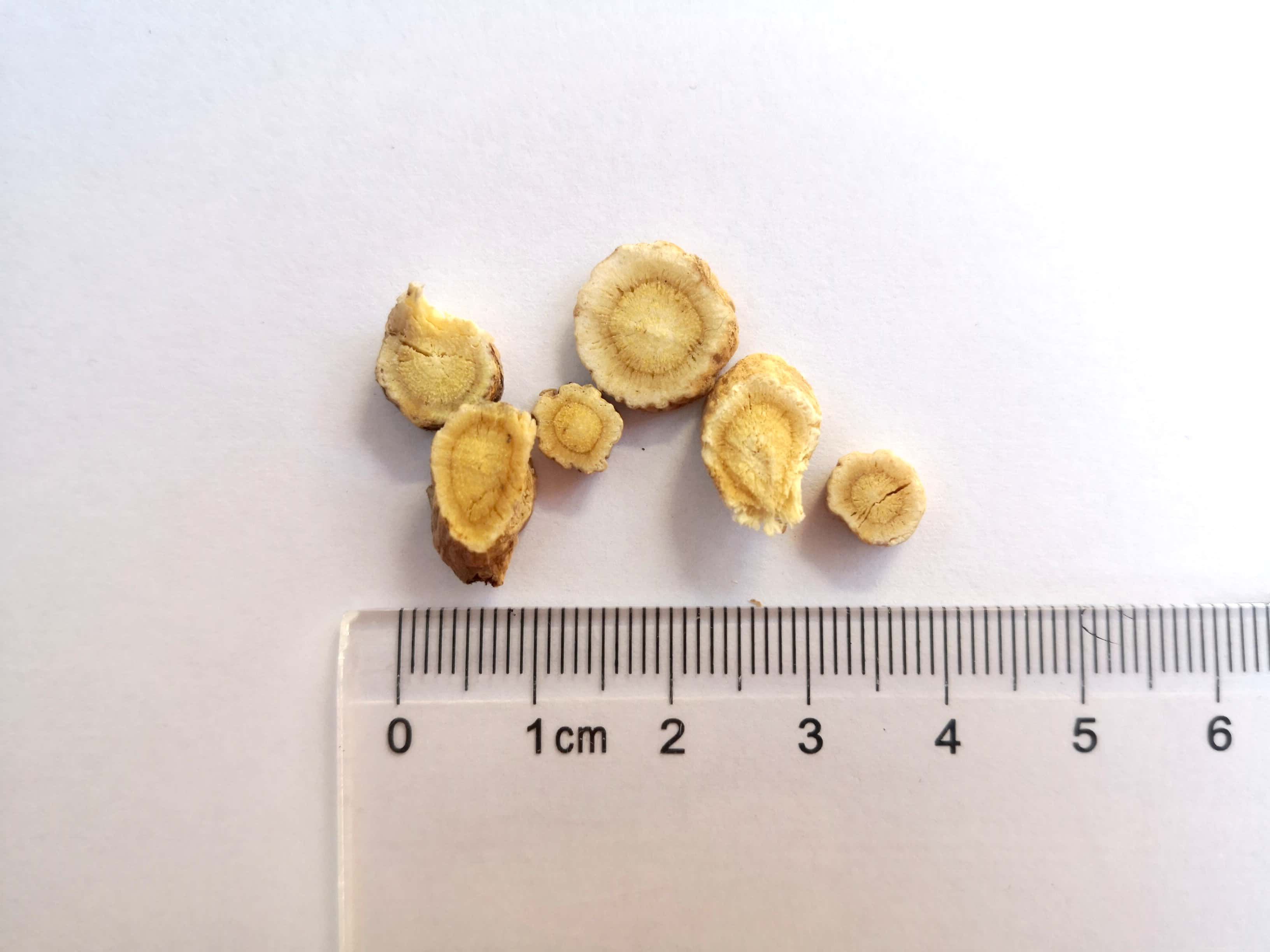 |
| QYDP19B | Redroot sage | 1912003 | Shanxi Tasly Plant Pharmaceutical Co., Ltd | 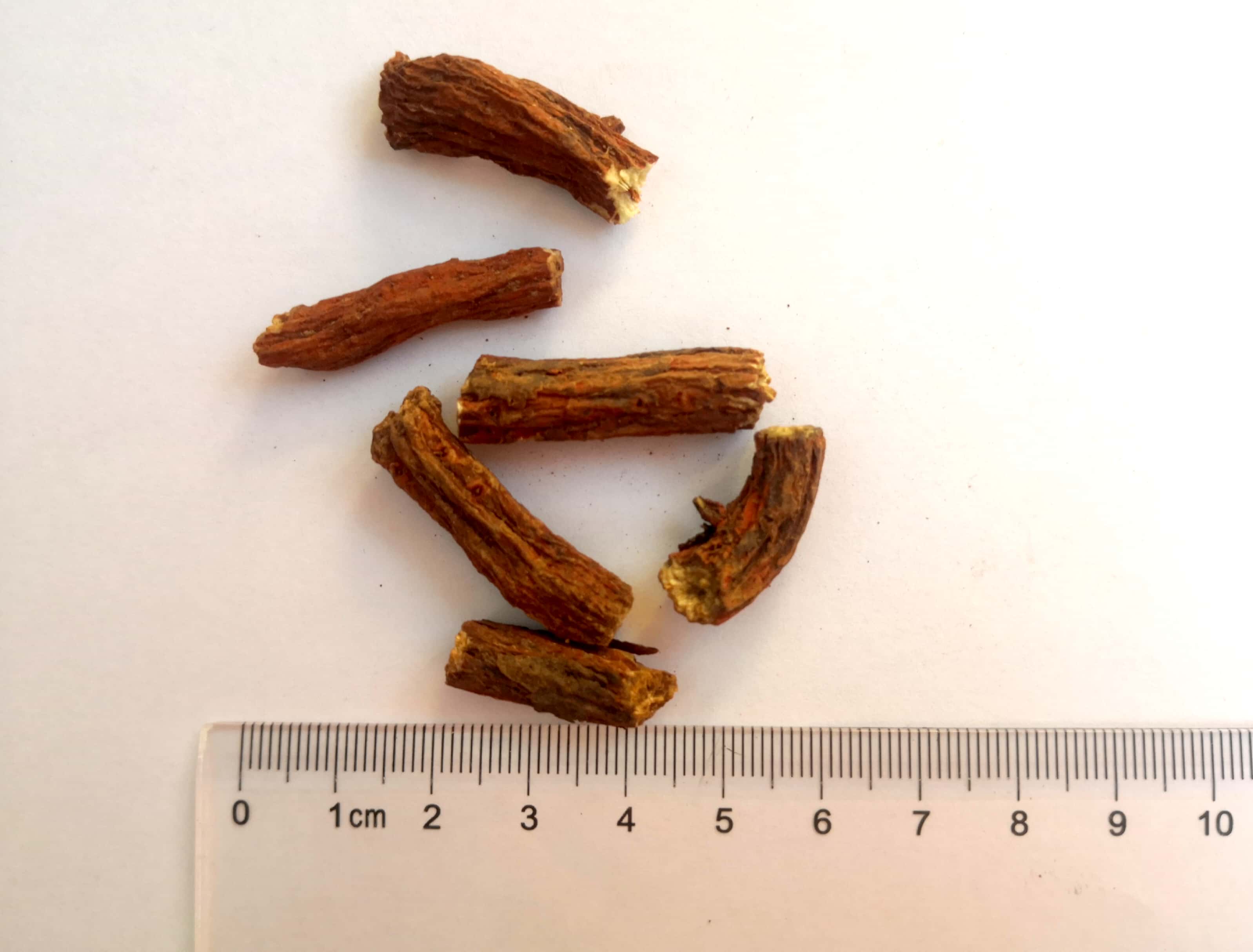 |
| QYDP19C | Pseudoginseng | 2002001 | Yunnan Tasly Notoginseng Pharmaceutical Co., Ltd | 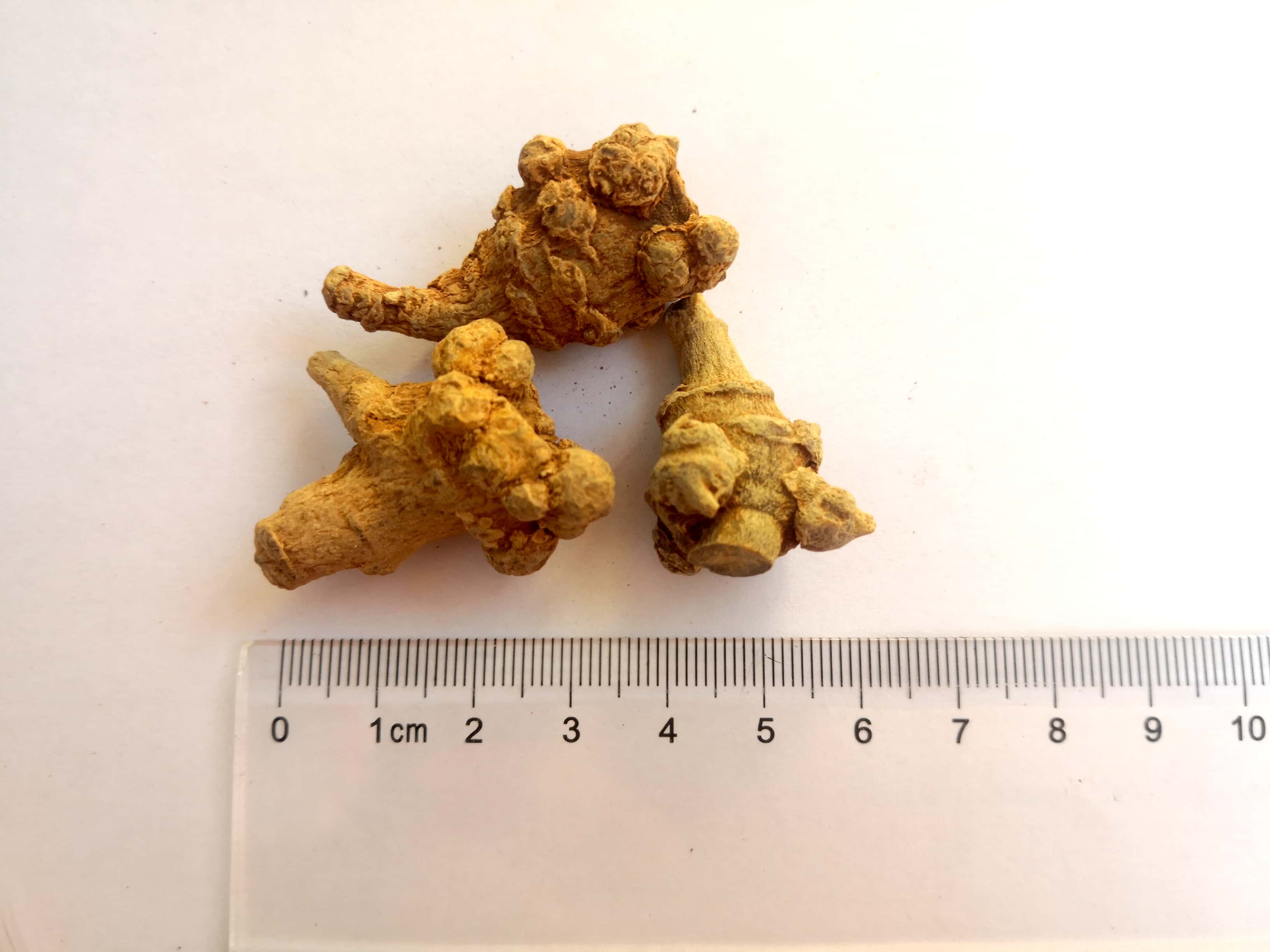 |
| QYDP19D | Fragrant rosewood | 2003001 | Anhui Yong Chinese Herbal Medicine Company Limited | 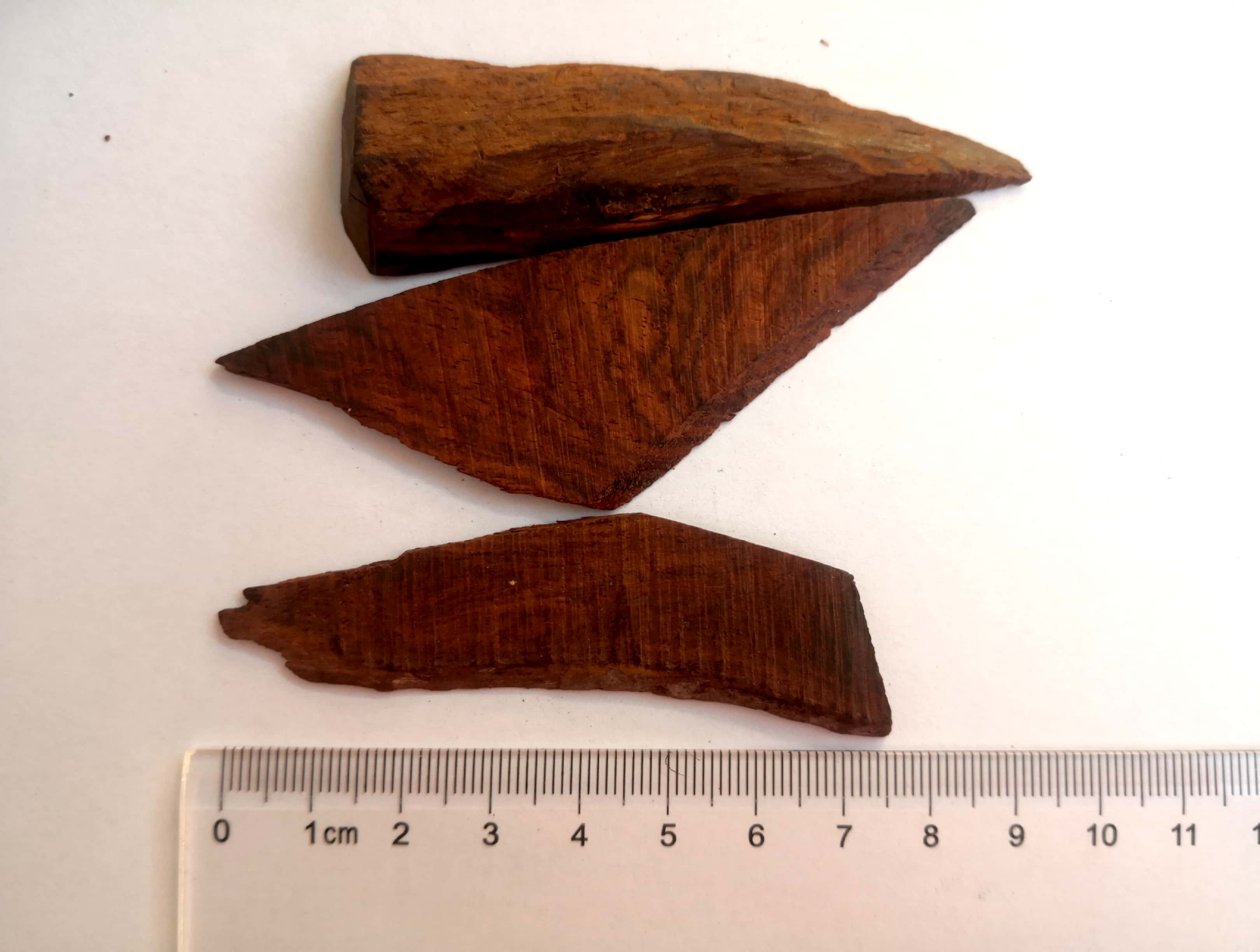 |
